# Exploratory study on the effect of orofacial myofunctional therapy on habitual tongue resting position in people with obstructive sleep apnea

**DOI:** 10.1590/2317-1782/e20250161en

**Published:** 2026-03-30

**Authors:** Natália de Castro e Silva Martins, Luciana Moraes Studart-Pereira, Hilton Justino da Silva

**Affiliations:** 1 Departamento de Fonoaudiologia, Universidade Federal de Pernambuco – UFPE - Recife (PE), Brasil.

**Keywords:** Speech, Language and Hearing Sciences, Myofunctional Therapy, Sleep Apnea, Obstructive, Tongue, Ultrasonography

## Abstract

**Purpose:**

To analyze the effect of OMT on the habitual tongue resting position and the results in people with OSA.

**Methods:**

This exploratory study comprising 18 individuals. The study considered eligible individuals of both sexes with a medical diagnosis of OSA (polysomnography) and orofacial myofunctional disorder. Exclusion criteria were those with a history of surgery for OSA or head/neck surgery, cancer in the region, previous oral motor speech-language therapy, psychiatric or neurological disorders that impair communication, oropharyngeal dysphagia, or neuromuscular diseases. They were submitted to speech-language-hearing assessment, sleep questionnaires (Pittsburgh and Epworth), anthropometric measurement, photo/film recordings, and tongue ultrasound examination. Individuals with an indication for OMT had 12 individual weekly sessions and were subsequently reassessed.

**Results:**

Changes were observed in mean tongue distances of the tongue regions pre- and post-OMT. The anterior region (RA) increased from 3.21 mm to 3.38 mm; the middle region (MR) increased from 3.14 mm to 3.25, and the posterior region (RP) decreased from 2.65 to 2.57. The analysis of delta percentage differences between tongue regions pre- and post-OMT shows that the mean percentage ± SD of AR increased by 3.5±6.1, and that of PR decreased by -3.8±4.8. The deltas of AR and PR were significantly different.

**Conclusion:**

OMT changes the distances between the base of the tongue and the contour surface of its three regions, increasing AR and decreasing PR, which generated subjective improvements in the sleep quality of patients with OSA, reflected in lower Pittsburgh and Epworth scores.

## INTRODUCTION

Sleep is extremely important for normal nervous system functioning. It is defined as a neurochemical process involving specific brain centers that makes one fall asleep and wake up. Studies show that a large part of the population has complaints regarding sleep, with a high prevalence of sleep disorders^(1-3)^.

Obstructive sleep apnea (OSA), characterized as sleep-disordered breathing (SDB), is one of the most studied sleep disorders and considered a public health problem, due to its high prevalence and mortality^(4,5)^. Research shows that OSA affects about 9% to 24% of adults and in Brazil is among the 10 countries with the highest estimated number of people with OSA, at 49 million individuals with an apnea-hypopnea index (AHI) ≥ 5 events/hr and 25 million with an AHI ≥ 15 events/hr^(4,5)^. In this regard, the Brazilian government created the National Policy for the Care of Sleep Disorders (Bill 496/24), whose main objective is to promote awareness, prevention, and treatment of sleep disorders. In addition, it aims to structure access to services, train professionals, and monitor the issue as a public policy^(6)^.

OSA is a multifactorial disorder with contributing phenotypes^(7)^, such as hypopnea episodes, defined as the reduced airflow signal amplitude ≥ 30% of the baseline, using nasal pressure, with oxygen desaturation ≥ 3% or associated with an awakening – or apnea, defined as the reduced airflow signal amplitude ≥ 90% for at least 10 seconds during sleep, due to upper airway narrowing, despite the maintenance of respiratory efforts^(7-10)^. It can lead to various morbidities, such as ischemia, hypertension, stroke, depression, insomnia, anxiety, and coronary heart disease, increasing the incidence of dementia syndromes, brain function disorders, and the risks of traffic and work accidents due to excessive daytime sleepiness^(10)^.

Positive pressure therapy is the gold standard treatment of choice for individuals with moderate to severe OSA^(11^. However, other therapies may be indicated, such as orofacial myofunctional therapy (OMT), which can be used alone or in combination to manage OSA. It can often reduce AHI in approximately 50% of patients with OSA, reduce snoring, improve sleep quality, increase oxygen saturation levels, and reduce daytime sleepiness^(12-15)^.

OMT uses exercises and other strategies to improve the sensitivity, proprioception, mobility, coordination, and strength of the oral and oropharyngeal structures to increase muscle tone, resistance, coordinated oropharyngeal muscle movements, and adequate breathing, chewing, swallowing, and speaking performance. Thus, it has a good potential as an alternative, noninvasive treatment of OSA^(12-15)^.

Studies on tongue behavior have been growing in oral motor therapy, the speech-language-hearing specialty that uses OMT. Hence, tongue ultrasound (US) is gaining visibility, as it provides dynamic and static images, and a computer program connected to the US helps select the frame that best represents the movement of the analyzed segment^(16^.

US has the advantages of being noninvasive, radiation-free, portable, and low-cost, providing high-resolution images of neck structures to visualize tongue movements in various planes. Therefore, this method has been used in the complementary diagnosis of OSA^(17,18)^.

Given the scarcity of research specifically addressing the habitual resting position of the tongue in individuals with OSA this research aimed to analyze the effect of OMT on the habitual resting position of the tongue and its impacts on OSA. Knowing the effect of OMT on the habitual tongue resting position in people with OSA will increase the knowledge of tongue behavior after a specific speech-language-hearing therapy approach and confirm the OMT benefits to these people’s oropharyngeal structure and OSA-related daytime signs.

## METHOD

The research project was submitted to the Human Research Ethics Committee, following resolution 466/12 of the National Health Council and approved under evaluation report number: 5.249.094 and was submitted and registered in the Brazilian Clinical Trials Registry (ReBEC) as a retrospective study. All participants signed an informed consent form before data collection. The study is a single-arm clinical trial with purposive sampling, conducted between August 2022 and June 2023.

**Figure 1:**
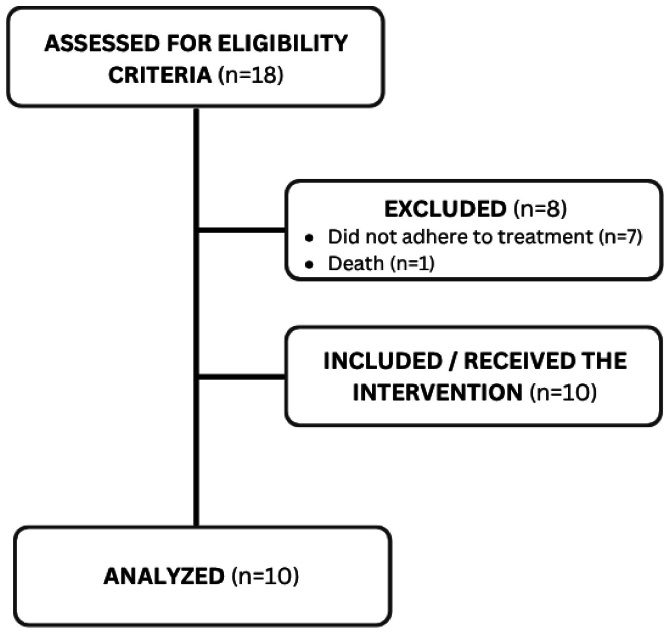
Study flow diagram according to the CONSORT guidelines, adapted for a single-arm clinical trial, showing the number of participants assessed, excluded, included, followed up, and analyzed

In a partnership with some centers for SDB study and diagnosis in Recife, 18 individuals met the inclusion criteria and were invited to participate in the research. This is an exploratory study and the process of participant recruitment, follow-up, and analysis are presented in the flow diagram, prepared according to the CONSORT guidelines for clinical trials, adapted for the single-arm format (Figure 1).

**Caption:** a – pre-OMT and b – post-OMT

The study considered eligible people of both sexes with a medical diagnosis of OSA (through polysomnography) and orofacial myofunctional disorder (OMD) (through the Orofacial Myofunctional Evaluation Protocol with Scores-extended [OMES-E], validated for the population with OSA^(18)^.

The research excluded those with a history of surgical intervention to treat OSA or previous head and neck surgery, previous or current diagnosis of head and neck cancer, previous history of oral motor speech-language-hearing therapy, diagnosis with a report of psychiatric or neurological disorders that hinder communication, oropharyngeal dysphagia, or neuromuscular diseases.

The collection was divided into three stages. In the first one, participants filled out a questionnaire with identification data and their general health conditions and submitted the polysomnography results and medical referral with an indication for OMT. Also, those who used *CPAP* or other treatments provided information about them.

Afterward, they filled out the Pittsburgh Sleep Quality Index^(19)^ and the Epworth Sleepiness Scale^(20)^ to investigate their sleep quality and daytime sleepiness.

Then, participants underwent individual anthropometric assessments to collect their weight and height, calculate their body mass index (BMI), and measure their neck circumference (NC) and abdominal circumference (AC). They were also classified according to the modified Mallampati classification, which importantly assesses other predictive factors of OSA severity^(21)^. NC measures > 38 mm for women and 40 mm for men were considered predictive of OSA^(22)^.

In this evaluation stage, each participant’s tongue was examined with US in its usual resting position. The submandibular region was cleaned by rubbing it with 70% alcohol and cotton. The subject was asked to swallow saliva before acquiring the images. Participants were instructed to remain silent and motionless and avoid moving their tongues or swallowing during the examination. The same experienced speech-language-hearing pathologist performed all US procedures.

The transducer was placed in the midline between the hyoid bone and the mandibular symphysis. A conductive gel was used for impedance coupling between the transducer surface and the skin surface of the participant's submandibular region, providing an image of the tongue surface in the sagittal plane. Two recordings were made, lasting around 20 seconds each. The evaluator held and tilted the transducer, varying the compressions for contact stability in the submental region until the edge of the tongue was visualized.

The examination was performed with Micro Ultrasound System – MicrUS EXT – 1H, manufactured by TELEMED, using a micro-convex transducer attached to a computer. US images were acquired with AAA (Articulate Assistant Advanced) software, version 2.17.02. Depth adjustments and image settings were personalized before recording. US images were acquired with an imaging frequency of 6.0 kHz, 120° imaging field, and 60 Hz sampling rate. Recorded data was saved individually and exported to image files.

The second stage of the study consisted of the OMT specifically for SDB based on scientific evidence^(23,24)^, guided by a flowchart developed by the researchers, lasting 12 weeks with individual 30-minute weekly sessions. This instrument was based on a set of exercises for breathing and for the structures involved in airway collapse, such as the tongue, soft palate, lateral pharyngeal walls, and orofacial function organization. This stage also had a generic approach with guidelines on sleep hygiene, general behavioral measures, and recumbency.

Orofacial myofunctional therapy was conducted in a protocol of 12 weekly sessions, with exercises individually selected and progressed according to each participant’s needs and myofunctional condition. In the first week, participants received guidance on sleep hygiene, the importance of healthy habits, and posture and positioning during sleep. In the second week, educational and awareness content was addressed regarding the physiology of stomatognathic functions (breathing, swallowing, and mastication) and nasal lavage. Between the third and ninth weeks, the focus was on training specific muscle groups (respiratory, perioral, oropharyngeal, and velopharyngeal) and improving stomatognathic functions, maintaining nasal lavage. During the final weeks (10th to 12th), emphasis was placed on exercise refinement, muscular endurance, and consolidation of the skills acquired.

At the end of each session, participants received written information and guidance on how they should perform the exercises on the other days of the week. They also received a form to check the activities done routinely at home – which, however, some participants did not submit to the researchers.

Immediately after the 12 intervention sessions, the third stage of the research had a complete reassessment, reapplying the initial protocols and questionnaires to analyze the same parameters as in the previous stages for subsequent comparative analysis. All participants were also reassessed with tongue US and polysomnography also after the completion of the OMT. However, in some subjects, reassessment with polysomnography lasted more than 4 months.

Data analysis was divided into some steps. The first one analyzed US records of the tongue and selected an image (frame) in AAA software. For this analysis, a thorough evaluation of several videos was carried out. Only one frame of the tongue at rest before and another one after OMT were selected per participant. The criterion for selecting these images was the visualization of the participant’s tongue contour from tip to root.

**Figure 2:**
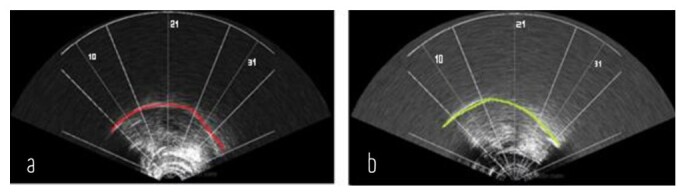
Splines of the contour frames on the tongue pre and post-orofacial myofunctional therapy in the sagittal plane

Then, the examinations were observed qualitatively. The frame with the best visualization of the tongue contour was highlighted and the splines (contour lines) were manually marked on the US image that had the entire tongue surface, using the drawing tool available in the AAA software. The splines in pre-OMT frames were indicated in red, and those in post-OMT ones were indicated in green (Figure 2).

After marking the splines, the distances in the contour of the tongue surface were analyzed in three portions (anterior, middle, and posterior regions). The portions of the tongue were determined after completely visualizing them from the anterior to the posterior region in front of the hyoid bone shadow. Measures were taken based on the software's predefined coordinates in the Spline workspace tab. The splines outlined in the selected frames were standardized with intrasubject comparative evaluation, thus pairing the regions under analysis. Hence, the entire contour of the tongue surface had to be visible in the chosen image.

The coordinates chosen were the number “10” for the anterior region of the tongue, “21” for the middle region, and “31” for the posterior region (Figure 2). The measures were taken from the center of distribution of the coordinate lines (defined as the zero point) to the point where the tongue contour crossed the chosen coordinate. For this phase, the examiner performed the measurements three times to certify the result. After checking this distance, the data were distributed in an Excel spreadsheet.

The analyses were performed in SPSS 26.0 (Statistical Package for the Social Sciences) for Windows and Excel 365. All tests were applied with a 95% confidence interval. Associations were verified with Fisher's Exact test for categorical variables; Wilcoxon (non-normal) and Spearman's coefficient test (non-normal) were applied between paired groups. The Shapiro-Wilk normality test was used for quantitative variables, and ANOVA with Tukey's post hoc (normal distribution) was used for comparisons with more than two groups.

## RESULTS

The study began with 18 participants. However, seven of them did not continue the speech-language-hearing rehabilitation process due to unavailability or reasons beyond their control, and one patient died due to other clinical health complications. Thus, 10 subjects – six men and four women aged 20 to 73 years – continued the research and had their data analyzed. Of these, seven had a bachelor’s degree, two had finished only elementary school, and one had no formal education. Moreover, only three had positive pressure therapy, whereas the other seven did not.

Table 1 presents the results of a comparison analysis between OMES-E variables and the adapted Mallampati classification, at two different moments, called "pre" and "post". Initially, 50% of the subjects had OMD and the other 50% had no relevant OMD. After OMT, 90% of patients were classified as having no OMD, while only 10% were still diagnosed with it. The modified Mallampati classification shows significant differences between the two moments, as the initial analysis classified nine subjects in class 4 and one in class 3, whereas after OMT five subjects were classified in class 1, three subjects in class 2, and two in classes 3 and 4.

**Table 1:** Comparison of orofacial myofunctional disorder, modified Mallampati classification, and subjective sleep indices (sleep quality and daytime sleepiness scale) before and after orofacial myofunctional therapy

**Variables**	**Moment**		**p-value** ^*^
	**Pre**	**Post**	
	**n (%)**	**n (%)**	
**OMES-E**			
With relevant OMD	5 (50.0)	1 (10.0)	0.141
Without relevant OMD	5 (50.0)	9 (90.0)	
**Mallampati**			
Class 1	0 (0.0)	5 (50.0)	**< 0.001**
Class 2	0 (0.0)	3 (30.0)	
Class 3	1 (10.0)	1 (10.0)	
Class 4	9 (90.0)	1 (10.0)	
**Pittsburgh**			
Good sleep quality	1 (10.0)	8 (80.0)	**0.005**
Poor sleep quality	9 (90.0)	2 (20.0)	
**Epworth**			
Normal sleep	1 (10.0)	4 (40.0)	**0.028**
Moderate sleepiness	0 (0.0)	3 (30.0)	
Excessive sleepiness	9 (90.0)	3 (30.0)	

(*) Fisher’s exact test

**Caption:** OMD – Orofacial myofunctional disorder

The comparison results of subjective sleep measure indices (sleep quality and daytime sleepiness scale) pre- and post-OMT are likewise presented in Table 1. There was a statistically significant difference in the prevalence of the variables “Pittsburgh” and “Epworth” concerning the moments analyzed, demonstrating that after OMT participants increased subjective sleep quality and decreased daytime sleepiness.

The comparative analysis of anthropometric characteristics and polysomnographic parameters of pre- and post-OMT patients are described in Table 2. It shows that the subjects maintained similar median BMI values pre (28.1 kg/cm^2^) and post (27.9 kg/cm^2^) (p value=0.594), NC values pre (37.5 cm) and post (37.6 cm) and p value=0.059, AC decreased significantly from pre (98.4 cm) to post (96.2 cm) (p value=0.011), AR pre (3.3 mm) and post (3.4 mm) (p value=0.011), and PR pre (2.7 mm) and post (2.6 mm) (p value =0.066). Despite not having a statistically significant difference, the median AHI decreased from 24.4 events/hr (initial) to 11.2 events/hr (final) (p value=0.051). A statistically significant difference was observed in ODI (p = 0.028), whose median decreased from 23.9 events/hr (pre-OMT) to 10.8 events/hr (post-OMT).

**Table 2:** Comparison of the patients’ anthropometric characteristics and polysomnographic parameters before and after orofacial myofunctional therapy

**Variables**	**Moment**		**p-value** ^*^
	**Pre-OMT**	**Post-OMT**	
	**Median (Q_1_; Q_3_)**	**Median (Q_1_; Q_3_)**	
BMI Kg/cm^2^	28.1 (23.9; 31.1)	27.9 (25.6; 30.5)	0.594
NC (cm)	37.5 (33.8; 41.1)	37.6 (33.8; 40.1)	0.059
AC (cm)	98.4 (94.4; 101.6)	96.2 (90.7; 99.3)	**0.011**
AHI (events/hr)	24.4 (8.0; 30.6)	11.2 (4.1; 22.1)	0.051
ODI (events/hr)	23.9 (10.7; 34.5)	10.8 (5.2; 20.2)	**0.028**
AR (mm)	3.3 (3.0; 3.5)	3.4 (3.1; 3.6)	0.139
MR (mm)	3.2 (2.9; 3.4)	3.2 (2.9; 3.4)	0.284
PR (mm)	2.7 (2.5; 2.8)	2.6 (2.4; 2.7)	0.066

(*) Wilcoxon test

**Caption:** BMI – Body mass index; NC – neck circumference; AC – abdominal circumference; AHI – apnea-hypopnea index; ODI – oxygen desaturation index; AR – anterior region; MR – middle region; PR – posterior region

Table 3 correlates several variables, highlighting the coefficients between them. The correlation between AHI, AR, MR, and PR pre- and post-OMT demonstrates significant coefficients between initial and final AHI (r = 0.800), pre and post AR (r = 0.782), pre AR and post MR (r = 0.697), post AR and post MR (r = 0.721), pre PR and post AR (r = 0.638), pre and post MR (r = 0.888), and pre PR and post MR (r = 0.809).

**Table 3:** Correlation between the apnea-hypopnea index and the distances between the base of the tongue and the contour surfaces of its three regions before and after orofacial myofunctional therapy

**Variables**	**Initial AHI**	**Final AHI**	**AR - Pre**	**AR - Post**	**MR - Pre**	**MR - Post**	**PR - Pre**
**Final AHI**	0.800 *	-	-	-	-	-	-
**AR – Pre**	0.503	0.400	-	-	-	-	-
**AR – Post**	0.139	-0.100	0.782 *	-	-	-	-
**MR – Pre**	0.243	0.109	0.748 *	0.596	-	-	-
**MR – Post**	0.152	0.033	0.697 *	0.721 *	0.888 *	-	-
**PR – Pre**	0.298	0.126	0.650 *	0.638 *	0.744 ^*^	0.809 *	-
**PR – Post**	0.274	0.025	0.268	0.189	0.477	0.427	0.428

Spearman’s correlation

(*) p-value ≤ 0.05

**Caption:** AHI – apnea-hypopnea index; AR – anterior region; MR – middle region; PR –posterior region

There were differences in the mean distances of the tongue regions pre- and post-OMT. Initially, AR was 3.21 mm, increasing to 3.38 mm post-OMT; MR went from 3.14 mm to 3.25 mm post-OMT; and PR went from 2.65 mm to 2.57 mm (Table 4).

**Table 4:** Mean distances of the tongue regions before and after orofacial myofunctional therapy

	**Moment**		**p-value**
	**Pre**	**Post**	
	**Mean (mm)**	**Mean (mm)**	
**AR**	3.21	3.38	0.139
**MR**	3.14	3.25	0.284
**PR**	2.65	2.57	0.066

**Caption:** AR – anterior region; MR – middle region; PR – posterior region

Table 5 shows the analysis results of the delta percentage difference between tongue regions pre-OMT and post-OMT. The mean percentage ± SD of AR increased by 3.5 ± 6.1, and that of PR decreased by -3.8 ± 4.8. Moreover, the delta of PR was statistically significantly different from that of AR.

**Table 5:** Comparison of differences of tongue regions in ultrasound assessment before and after orofacial myofunctional therapy

**Variables**	**Mean ± SD**	**p-value** ^*^
**Ultrasound deltas (%)**		
AR	3.5 ± 6.1	0.009*
MR	1.6 ± 4.0	
PR	-3.8 ± 4.8^A^	

A Statistically significant difference in relation to AR

(*) p-value ≤ 0.05

**Caption:** AR – anterior region; MR – middle region; PR – posterior region

## DISCUSSION

This study had a prevalence of males, aged 20 to 59 years. This finding corroborates other studies, which had a predominance of males with OSA, whose incidence increased with age and BMI^(24)^.

This research found that OMT reduced ODI and AHI pre- and post-OMT. Also, 90% of the subjects did not have OMD after OMT. During the period, the subjects maintained the same median BMI and NC, further highlighting the impact of OMT on the study population. These findings corroborate other studies, which indicate that OMT can reduce AHI by approximately 50% in adults. Hence, it is an auxiliary OSA treatment in individuals with primary snoring or mild to moderate OSA, since oropharyngeal exercises effectively change the tongue muscle tone, reduce SDB symptoms and mouth breathing, and increase oxygen saturation. It also provides benefits such as improved quality of life and greater adherence to CPAP^(23-26)^.

Individuals with OSA tend to have myofunctional changes – the greater the degree of orofacial structure impairment, the greater the degree of OSA severity. Therefore, OMT is even more important in cases of OSA, as it helps organize oropharyngeal structures and functions^(25)^.

This research showed a significant association in the Mallampati classification after the intervention, indicating it as an important predictive factor for reducing OSA severity, since the greater the disproportion of the oral cavity anatomy, the greater the risk for OSA^(27)^.

In the present study, a significant reduction in AC was observed after the intervention. This finding is consistent with a recent study reporting that AC is a significant predictor of OSA severity in adults^(28)^. However, despite the significant reduction in AC, this change was not accompanied by a substantial decrease in BMI, suggesting that the finding may not have translated into clinically relevant changes in the respiratory parameters evaluated by polysomnography.

Research shows that reduced pharyngeal air space, inferiorly positioned hyoid bone, and increased tongue area and length can compromise upper airway dimensions in patients with OSA since these structures play an important role in the physiological maintenance of the upper airway^(29)^. The compared distances between the tongue regions and base in this study showed that AR was more distant, and PR was less distant from the base post-OMT. This highlights the important role of OMT in adapting tongue resting position in people with OSA, which can have positive results in reducing upper airway obstruction during sleep. Although these changes occurred on a millimeter scale, they were accompanied by reductions in important polysomnography parameters, such as AHI and ODI. This highlights the important role of OMT in adapting the tongue's resting position in individuals with OSA, contributing to the reduction of upper airway obstruction during sleep.

Tongue US provides a better understanding of the impacts of OMT on this structure. Studies that used tongue US to assess patients with OSA indicate an association between the distances of the tongue arteries at the base of the tongue and the existence and severity of OSA, highlighting that distances of 30 mm can be considered a risk factor for moderate to severe OSA. Furthermore, the thickness of the base of the tongue measured with submental US has been widely used to predict OSA severity^(30)^.

This research demonstrated a significant improvement in subjective sleep quality and excessive sleepiness. Those surveyed must have observed this improvement due to the decreased ODI (which had a significant association) and AHI (which, despite not having an association, decreased by almost 50%). This may be due to the adequate tongue resting position during sleep and the consequent decrease in SDB. Another study shows that OMT reduces daytime sleepiness and significantly improves subjective sleep quality^(23)^.

This study has some limitations, such as the few study subjects and those lost in the process; the lack of a control group and randomization, the possible interference in tongue position as the transducer is handled differently during US assessments; is the absence of blinding in the analyses; the time between the assessment and reassessment in some cases and the patients did not present the same characteristics and some were exposed to CPAP treatment. As a limitation, we also highlight the lack of inter-examiner reproducibility.

Nonetheless, this exploratory study obtained objective data on the impact of OMT on individuals with OSA. It also demonstrated the use of US as an important aid to diagnose and monitor patients with OSA. Further research is suggested, evaluating more people to investigate the tongue resting position in people with OSA and the possibility of introducing tongue US to assess patients with OSA in daily practice.

## CONCLUSION

OMT satisfactorily reduced OMD and improved sleep quality and excessive sleepiness.

OMT decreased AHI and ODI, although their coefficients were not significantly related to the tongue regions. On the other hand, ODI was significantly associated before and after OMT.

The distances between the base of the tongue and the contour surface of its three regions changed after OMT. The distance of AR and MR increased and that of PR decreased. Furthermore, the increase in AR was correlated with the decrease in PR.

Hence, OMT stands out as a possible efficient treatment in cases of OSA, and tongue US can be considered an important tool to assess these patients as a complementary option in clinical and polysomnographic evaluation.
